# When less is more: reducing redundancy in mental health and psychosocial instruments using Item Response Theory

**DOI:** 10.1017/gmh.2019.30

**Published:** 2020-01-09

**Authors:** Emily E. Haroz, Jeremy C. Kane, Amanda J. Nguyen, Judith K. Bass, Laura K. Murray, Paul Bolton

**Affiliations:** 1 Department of International Health, Johns Hopkins Bloomberg School of Public Health, Baltimore, Maryland, USA; 2 Department of Epidemiology, Columbia University Mailman School of Public Health, New York, New York, USA; 3 Curry School of Education, University of Virginia, Charlottesville, Virginia, USA; 4 Department of Mental Health, Johns Hopkins Bloomberg School of Public Health, Baltimore, Maryland, USA; 5 Center for Humanitarian Health, Johns Hopkins Bloomberg School of Public Health, Baltimore, USA

**Keywords:** Instruments, Item Response Theory, measurement

## Abstract

**Background:**

There is a need for accurate and efficient assessment tools that cover a range of mental health and psychosocial problems. Existing, lengthy self-report assessments may reduce accuracy due to respondent fatigue. Using data from a sample of adults enrolled in a psychotherapy randomized trial in Thailand and a cross-sectional sample of adolescents in Zambia, we leveraged Item Response Theory (IRT) methods to create brief, psychometrically sound, mental health measures.

**Methods:**

We used graded-response models to refine scales by identifying and removing poor performing items that were not well correlated with the underlying trait, and by identifying well-performing items at varying levels of a latent trait to assist in screening or monitoring purposes.

**Results:**

In Thailand, the original 17-item depression scale was shortened to seven items and the 30-item Posttraumatic Stress Scale (PTS) was shortened to 10. In Zambia, the Child Posttraumatic Stress Scale (CPSS) was shortened from 17 items to six. Shortened scales in both settings retained the strength of their psychometric properties. When examining longitudinal intervention effects in Thailand, effect sizes were comparable in magnitude for the shortened and standard versions.

**Conclusions:**

Using Item Response Theory (IRT) we created shortened valid measures that can be used to help guide clinical decisions and function as longitudinal research tools. The results of this analysis demonstrate the reliability and validity of shortened scales in each of the two settings and an approach that can be generalized more broadly to help improve screening, monitoring, and evaluation of mental health and psychosocial programs globally.

## Background

Interest in the provision of mental health and psychosocial interventions for populations in low- and middle-income countries (LMIC) has dramatically increased over the past decade. The proliferation of studies measuring prevalence of mental health problems and evaluating the impact of treatment approaches in LMIC (van Ginneken *et al*., 2013; Jordans *et al*., 2016; Singla *et al*., 2017; Yatham *et al*., 2017) underscores the need for accurate and efficient tools that assess multiple mental and psychosocial problems and related outcomes (e.g. functional impairment, social support).

Presently, self-report measurement instruments predominate in both research studies and treatment settings (Smits *et al*., 2007). This is particularly the case in LMIC where a shortage of trained mental health professionals precludes the use of diagnostic interviews or professional evaluations (World Health Organization, 2011). Comprehensive self-report assessments to measure multiple outcomes can result in lengthy assessment batteries that cause an undue burden on participants and may reduce accuracy as a result of respondent fatigue (Smits *et al*., 2007; De Vet *et al*., 2011; Smits and Finkelman, 2014). Perhaps even more critical, long assessments are not implementable, feasible, or sustainable in routine practice, leading to little to no use of valid standardized assessments among service providers in LMIC. Although a few exceptionally short instruments exist for initial screening (i.e. PHQ-2; two items) (Arroll *et al*., 2010), there are few that can be combined to measure a range of outcomes of interest, and used for a variety of purposes – screening, research, and clinical tracking. There is an unmet need for freely available, brief instruments that maintain or even improve the accuracy of standard measures for survey research, treatment planning, and evaluation of how people change as a result of mental health and psychosocial interventions.

This paper describes an innovative approach using Item Response Theory (IRT) analysis to develop short, pragmatic instruments that address the measurement challenges described above. While IRT has been used in scale-refinement in previous studies, we were unable to find articles that described this process using data from LMIC and community-based settings. We focused on scales measuring depression and posttraumatic stress symptoms as these are two of the most common mental disorders found worldwide (World Health Organization, 2017). Our process involved shortening longer scales that had previously been tested and found valid and reliable in each study setting. Our intent for this paper is to provide a template for a process of scale refinement – taking previously tested longer instruments and using IRT to select the best performing items to generate brief locally reliable and valid scales that measure multiple domains. A secondary goal of the paper was to produce shortened valid and reliable scales for the two study settings that can be used for future research and/or program monitoring and evaluation.

Using data from two different cultural contexts representing a sample of adults in Thailand and a sample of adolescents in Zambia, we aimed to determine whether we could generate shortened symptom measures that perform comparably to the standard ones that were longer in length. The goal of these analyses was to create measures that could be: (1) efficiently integrated and feasibly utilized in routine clinical care, and (2) used for longitudinal evaluation of psychotherapy interventions. While the results are limited to psychotherapy research in two settings, the approach could be generalized more broadly to improve screening, monitoring, and evaluation of mental health and psychosocial programs globally.

## Methods

### Data sources

Data are from studies utilizing scales for common mental health problems among populations in two LMIC settings. The first study uses data collected as part of a randomized controlled trial (RCT) of the Common Elements Treatment Approach (CETA) among Burmese adult refugees in Thailand (Bolton *et al*., 2014). For the current analysis, we analyzed data from *N*  =  653 participants who were screened for the RCT. We used IRT to generate shortened scales and then tested how well the shortened scales measured change over time in the enrolled sample (*N*  =  347). The second source of data is from HIV-affected adolescents (ages 13–17) in Zambia who completed assessments either as part of a cross-sectional instrument development study (*N*  =  210) (Kane *et al*., 2018; Murray *et al*., 2018*b*) or during screening for an RCT of trauma-focused cognitive behavioral therapy (*N*  =  610; NCT02054780) that used the measures tested in the instrument development study. These samples were selected due to their purposive sampling approach, which was intended to include people with and without mental health problems representing a range of underlying disease severity.

The study in Thailand was approved by the Johns Hopkins Bloomberg School of Public Health Institutional Review Board and a community ethnics board at the Mae Tao Clinic. All participants were 18 years old or older and provide informed consent. The Zambia studies were approved by the Johns Hopkins Bloomberg School of Public Health Institutional Review Board and the University of Zambia Ethics Committee. All participants were under 18 years old and provide informed assent. Parental/caregiver permission was obtained for all participants.

### Instruments

In Thailand, we analyzed data from two of the assessment measures: (1) The Hopkins Symptom Checklist 25 subscale for depression symptoms only (HSCL; 15 items) (Mollica *et al*., 2004); and (2) The Harvard Trauma Questionnaire (HTQ, 16 items) for symptoms of posttraumatic stress (Mollica *et al*., 2004). These measures were previously adapted and validated locally in the same context (Haroz *et al*., 2014). Items were measured using a Likert-type scale with response options ranging from 0 ‘none of the time’ to 3 ‘almost all the time.’ A total score is calculated by taking the mean of all responses. Recall period was set at 2 weeks. Results from the previous validation study indicated a single-factor structure for both scales, and good reliability (HSCL: Cronbach's *α*  =  0.92, test–retest reliability  =  0.84; HTQ: Cronbach's *α*  =  0.92, test–retest reliability  =  0.78) (Haroz *et al*., 2014). The HSCL-25 and HTQ were administered in full to the screening sample and again at follow-up for RCT participants.

The HSCL is one of the more widely used measures in global mental health and includes items related to both the Diagnostic and Statistical Manual's diagnostic criteria for Major Depressive Disorder (American Psychiatric Association, 2013), as well as, other symptoms commonly found in LMIC but not included in current diagnostic criteria (Haroz *et al*., 2016, 2017). While the Patient Health Questionnaire 9 (PHQ-9) is perhaps more commonly used, there is evidence to suggest that the PHQ-9 may be inferior at capturing how depression is expressed in many LMIC settings (Haroz *et al*., 2016, 2017).

We also included a local measure of functional impairment. This instrument was developed based on qualitative findings using a process described by Bolton and Tang (2002). The function instruments contained 16 items for men and 23 items for women due to men and women having different functional tasks in this context. Respondents were asked how much difficulty they had with each activity listed in the prior 2 weeks. Response options ranged from 0 ‘no difficulty at all’ to 4 ‘often cannot do.’ The male and female versions of the instruments showed excellent internal consistency reliability (*α*  =  0.91 and *α*  =  0.92, respectively) and test–retest reliability (*r*  =  0.89 and *r*  =  0.86, respectively) (Haroz *et al*., 2014).

In Zambia, the analysis was conducted on the Child PTSD Symptom Scale (CPSS) (Foa *et al*., 2001), a 17-item scale of pediatric trauma symptoms with a past-2-week reference period. Items were measured using a Likert-type scale with response options ranging from 0 ‘not at all’ to 3 ‘almost always.’ Participants completed the CPSS via Audio Computer Assisted Self-Interviewing (ACASI) (Kane *et al*., 2016). Scores were calculated by taking the mean of all responses for each participant. Results from a previously conducted instrument development and validation study with the CPSS in Zambia found that the measure had good internal reliability (Cronbach's *α*  =  0.93), adequate test–retest reliability (0.68), and strong criterion validity (the measure significantly discriminated between PTSD cases and non-cases at *p* < 0.05) (Murray *et al*., 2018*b*).

### Analysis

Item Response Theory (IRT) is a latent variable approach that models the probability of a given response as a function of a respondent's underlying level of a latent trait (Embretson, 1996; Hays *et al*., 2000). IRT can be used to refine scales by identifying poor performing items that are not well correlated with the underlying trait and can be removed to shorten scales. IRT can also be used to identify well-performing items at varying levels of a latent trait to assist in screening or monitoring purposes and prevent floor and ceiling effects (Edelen and Reeve, 2007). In addition, IRT can be used to identify where along with a latent trait a scale is under-performing and where additional items are needed in order to better assess individuals at those levels. Finally, IRT methods can identify where along the latent trait continuum, there is an excess of items, meaning redundant items measuring the same level of the underlying latent trait with the same level of accuracy of which some can be removed.

Due to the nature of the underlying data (i.e. one cross-sectional and from an instrument testing study, one longitudinal and from an intervention trial), in each sample, we were unable to conduct the same analyses in each sample. Thus, we provide methods and analyses separate for both the Thai and Zambian samples (see also Online Supplementary Table S1). However, by conducting different analyses in the different samples, we hope to illustrate how these methods can be applied and used for a variety of purposes.

#### Thailand

For the Thailand data, our analysis plan used several steps: First we randomly split the screening and baseline data (*N*  =  653) into a *development* sample and *test* sample using a one-half to one-half split as is common practice in scale refinement methods (Edelen and Reeve, 2007; Xia *et al*., 2019). In the development sample, we examined the dimensionality of each scale using Principal Components Analysis (PCA). We then fit separate unidimensional or multidimensional graded response models (GRMs), depending on the dimensionality indicated in the PCA, for each of the scales of interest. GRMs are a variation on the two-parameter logistic model and were selected based on the ordered nature of response categories (Samejima, 2016). Each model yields a discrimination parameter and multiple location parameters for each item. Item discrimination parameters are analogous to factor loadings and indicate how strongly an item is associated with the underlying latent trait. Generally, item discrimination values of 0.01–0.34 are considered very low; 0.35–0.64 low; 0.65–1.34 moderate; 1.35–1.69 high; and 1.70 and above, very high (Baker and Kim, 2004). An item location parameter (*b*) or item difficulty parameter is the point along the latent trait continuum (e.g. depression severity) at which the probability of endorsing a response at that level or lower is 50% (Baker and Kim, 2004). Examination of item location parameters allows the assessment of which items best measure different levels of severity as an endorsement of the item reflects that level of severity in the underlying trait. All IRT analyses were done using the screening and baseline data included in the training sample only.

Items for retention were selected based on five considerations: (1) high discrimination; (2) location parameters that represented a wide range of the latent trait; (3) reliability of item responses (if possible); (4) overlap with items on other scales (i.e. problems with sleep being relevant to both depression and PTSD); and (5) clinical relevance and utility as determined by a team of clinicians guiding the implementation of a clinical intervention. Choosing items based on these considerations would, in theory, produce a shortened assessment that would be able to measure low, moderate, and high levels of the latent traits while maintaining reliability and validity similar to that of the original scale. Selection of items was based on their performance in the *development* sample only. While we balanced these considerations in our selection of items, local context may dictate which of the considerations to weigh more heavily. For example, if there is an item that is particularly meaningful in a certain setting, this item may need to be retained regardless of its other properties.

Once items were selected, we used the *test* sample to (1) evaluate the internal consistency of the scales using Cronbach's *α*; (2) examine score distributions; and (3) calculate correlations between scale scores based on our nomological network as a measure of convergent validity (e.g. association of symptom scores and functioning) using the baseline data (*N*  =  181). We were unable to examine criterion validity as this baseline sample did not include a criterion. Using follow-up data from the Thailand RCT that was included in the test sample (*N*  =  181; Bolton *et al*., 2014), we calculated intervention effect sizes (Cohen's *d*) using the scale scores derived from the shortened instrument and compared these to the effect sizes calculated in the original RCT analysis (*N*  =  347) using the full scales.

#### Zambia

For Zambia, participants in each study were distinct but drawn from the same source population of adolescents who exhibited HIV risk behaviors and met the WHO criteria for orphan or vulnerable child (Kane *et al*., 2018; Murray *et al*., 2018*b*). Item selection for the CPSS based on GRMs was conducted with a random sample of 50% of the data from the instrument validation study. The original purpose of the validation study was to evaluate the psychometric properties and criterion validity of the CPSS and other measures that were intended for use in an upcomingRCT. We based item selection on the same considerations as above for Thailand. Using the other half of the instrument validation study data (Murray *et al*., 2018*b*), we compared the performance of a shortened CPSS to the original version across a number of psychometrics: (1) Cronbach's *α*; (2) score distributions (i.e. means and standard deviations); (3) criterion validity comparing average scores on the original CPSS and short CPSS for PTSD cases and non-cases (Kane *et al*., 2018; Murray *et al*., 2018*b*); and (4) clinical utility comparing Area Under the Curves (AUCs) using our criterion. We tested the shortened CPSS performance in the baseline RCT data by comparing the relative strength of correlations between the shortened CPSS version and the full version with three external measures: a locally-developed scale of functional impairment (Murray *et al*., 2015), and the Youth Self Report (YSR), which includes sub-scales of both internalizing and externalizing symptoms (Achenbach, 1991). In a validation study with the YSR in Zambia, we found it had strong psychometric properties for internal reliability (Cronbach's *α*  =  0.93 and 0.94, respectively, for internalizing and externalizing symptoms) and good criterion validity (Murray *et al*., 2018*b*). We hypothesized that the CPSS scales (both the full and shortened versions) would have significant positive correlations with all three external measures.

Parent studies were approved by both the Johns Hopkins Bloomberg School of Public Health Institutional Review Board and local review boards. The research presented in this paper is a secondary data analysis of de-identified data.

## Results

**Fig. 1.**
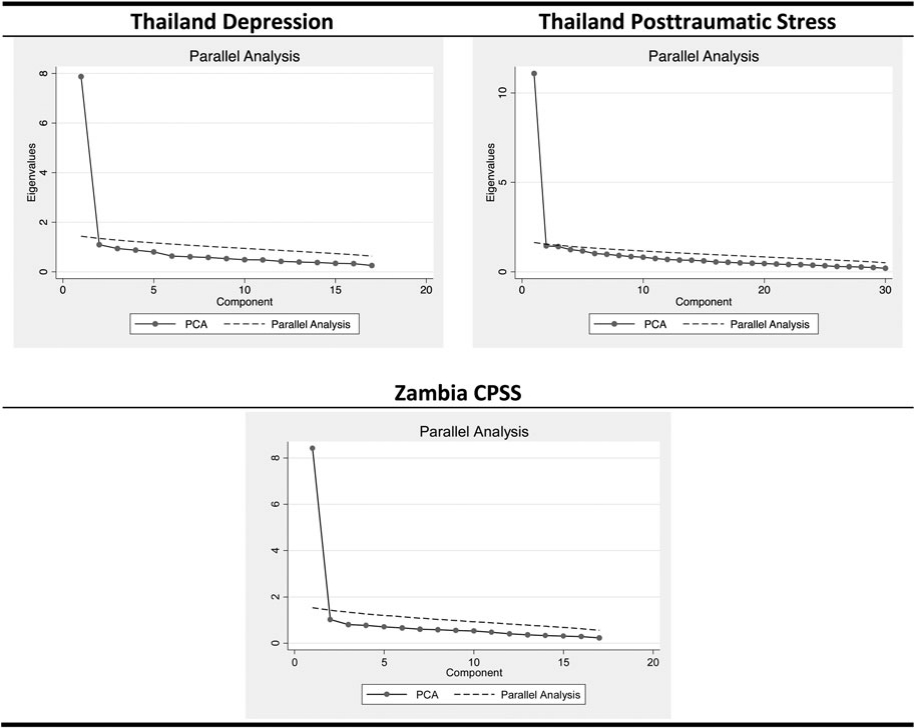
Principal Components Analysis (PCA) for items on original scales in Thailand and Zambia.

Demographic characteristics for the Thailand and Zambia study populations are provided in Table 1. PCA conducted on the Thailand and Zambia samples indicated predominantly unidimensional traits (unidimensionality is a key assumption of IRT models) for both depression (Thailand) and Posttraumatic Stress Scale (PTS) (Thailand and Zambia) (Fig. 1). In Zambia, three items were dropped from the original CPSS scale because of high uniqueness (>0.50): ‘upsetting thoughts/images,’ ‘not feeling close to those around you,’ and ‘overly careful.’

**Table 1.** Sample characteristics

	Thailand (*N* = 653)		Zambia (*N* = 210)	
Female; *N*, %	362	55.5	118	56.2
Age; Mean (s.d.)	35.2	11.9	14.9	1.4
Education status				
None	42	6.4	0	0.0
Primary	160	24.5	102	48.6
Middle	186	28.5	65	31.0
High school	135	20.7	32	15.2
More than high school	130	19.9	0	0.0
Other			11	5.2
Depression;^a^ Mean (s.d.), Range	0.86 (0.6)	0–2.8	–	
PTS scores;^a^ Mean (s.d.), Range	0.68 (0.5)	0–2.6	0.89 (0.7)	0–3
Function scores;^a^ Mean (s.d.), Range	0.94 (0.7)	0–3.5	1.2 (1.0)	0–4
Youth self-report: internalizing;^a^ Mean (s.d.), Range	–	–	21.3 (14.5)	0–64
Youth self-report: externalizing;^a^ Mean (s.d.), Range	–	–	15.6 (13.4)	0–64

a Depression, anxiety, PTS and function scored as averages

In Thailand, after fitting a GRM, item discrimination parameters ranged from *a*  =  1.03 for the item ‘Loss of sexual interest or pleasure’ to *a*  =  2.85 for the item ‘disappointed.’ Items that were indicative of measuring more severe depression (i.e. with higher difficulty parameters) included ‘Loss of sexual interest or pleasure,’ ‘Thoughts of ending your life; commit suicide,’ and ‘Feelings of worthlessness; no value.’ In Zambia, for the CPSS, item discrimination parameters ranged from *a*  =  1.78 for the item ‘bad dreams/nightmares’ to *a*  =  3.15 for the item ‘feelings in your body when thinking about the event.’ Items with higher difficulty parameters included ‘upset thinking or hearing about the event,’ ‘trouble falling or staying asleep,’ and ‘felt like the event was happening again.’ Full item parameter results are included as Supplementary material.

Tables 2 and 3 show the final items that were selected for each shortened scale. In Thailand, the original 17-item depression scale was shortened to seven items and the 30-item PTS was shortened to 10 items. The depression scale has four unique items, the PTS scale contains 10 unique items, and three additional items contribute to scoring for both scales. These three items were common to the HSCL and the HTQ although worded slightly differently on each scale (i.e. ‘Difficulty concentrating’ – same on both HSCL and HTQ; ‘Feeling no interest in things’  =  HSCL and ‘Less interest in daily activities’  =  HTQ, ‘Don't talk to anyone’[local phrasing of the item]  =  HSCL, and ‘Feeling detached or withdrawn from people’  =  HTQ). In Zambia, the CPSS scale was shortened from 17 items to six. The retained items represent a range of symptoms across the disorders, supporting the content validity of the shortened scales. For example, the depression scale includes assessment of depressed mood (e.g. ‘feeling sad; unhappy’) and loss of interest; the PTS scale includes symptoms related to re-experiencing, arousal, and negative thoughts; and the CPSS scale assesses sleep and concentration problems as well as loss of interest and problematic responses to traumatic memories.

**Table 2.** Items selected for shortened scales in Thailand

Depression	PTS
Feeling sad; unhappy	Recurrent thoughts or memories of the most hurtful or terrifying events
Feeling no interest in things^a^	Feeling as though the event is happening again
Feelings of being trapped or caught^a^	Sudden emotional or physical reaction when reminded of the most hurtful or traumatic events
Worry too much about things	Unable to feel emotions
Blaming self for things	Difficulty concentrating
Don't talk to anyone^a^	Feeling that people do not understand what happened to you
Disappointed	Feeling guilty for having survived
	Spending time thinking about why these events happened to you
	Feeling as if you are going crazy
	Feeling that you have no one to rely on

a Item is used in scoring of both depression and PTS scale scores.

**Table 3.** Items selected for shortened CPSS in Zambia

PTS
Nightmares/bad dreams
Upset thinking or hearing about event
Feelings in body when you think about the event
Less interest in doing things you used to do
Trouble falling or staying asleep
Difficulty concentrating

Table 4 shows the psychometric evaluation and utility comparing the standard scales to the shortened versions. In the full Thailand trial data reported elsewhere (Bolton *et al*., 2014), the standard depression and PTS scales had Cronbach's *α*  =  0.93 and *α*  =  0.95, respectively. The shortened versions when tested in half the enrolled sample performed similarly with *α*  =  0.90 for the short depression measure and *α*  =  0.92 for the short PTS measure. Ranges and standard deviations were larger for the shortened versions, indicating slightly less precision. Correlations of both standard and short scales to functional impairment were poor [Standard: *r*  =  0.17 and *r*  =  0.32 for depression and PTS (from original trial data); Short: *r*  =  0.10 and *r*  =  0.35 for depression and PTS (from testing sample of enrolled participants)]. Finally, effect sizes were comparable in magnitude between the effect measured in the original trial (Bolton *et al*., 2014) and those we obtained through testing in the random sub-sample of enrolled participants.

**Table 4.** Psychometrics of short *v.* long scales

	*α*	Mean at baseline (s.d.); Range	Effect size (Thailand only) (enrolled sample)				Correlations (construct validity)		
							Functioning	Internalizing	Externalizing
Thailand									
Depression									
Standard (*N* = 347; 17 items)	0.93	1.32 (0.17); 0–2.82	*d* = 1.16				*r* = 0.17	–	–
Short (*N* = 181; 7 items)	0.90	1.60 (0.45); 0–2.86	*d* = 1.31				*r* = 0.10	–	–
PTS									
Standard (*N* = 347; 30 items)	0.95	1.05 (0.37); 0–2.63	*d* = 1.19				*r* = 0.32	–	–
Short (*N* = 181; 13 items)^a^	0.92	1.08 (0.39); 0–2.37	*d* = 0.99				*r* = 0.35	–	–
Zambia			Criterion validity (Zambia only)						
CPSS			Case mean	Non-case mean	*p*	AUC			
Standard (17 items)	0.93	0.89 (0.7); 0–3	1.09 (0.8)	0.55 (0.6)	<0.0001	0.73	0.47	0.60	0.50
Short (6 items)	0.85	0.89 (0.8); 0–3	1.07 (0.8)	0.57 (0.6)	<0.0001	0.70	0.44	0.57	0.47

a Ten unique items; three items overlap with depression; total items for depression and PTS  =  17.

In Zambia, the Cronbach's *α* for the shortened six-item CPSS (*α*  =  0.85) was comparable to the full 17-item version (*α*  =  0.93). The means and ranges of the two versions were identical (0.89; 0–3); the standard deviation for the shortened scale (0.8) was slightly larger than the full version (0.7). Table 4 also shows criterion validity of the six-item CPSS. The shortened scale significantly (*p* < 0.0001) discriminated between psychosocial ‘cases’ (mean  =  1.07, s.d.  =  0.8) and ‘non-cases’ (mean  =  0.57, s.d.  =  0.6) in our validity data. Its discriminatory ability was similar to the full CPSS (‘cases’ mean  =  1.09, s.d.  =  0.8; ‘non-cases’ mean  =  0.55, s.d.  =  0.6, *p* < 0.0001). The AUCs were also similar between the six-item (0.70) and the 17-item (0.73) versions.

Using the RCT data, correlations between the shortened CPSS and functional impairment, internalizing, and externalizing were *r*  =  0.44, 0.57, and 0.47, respectively, which were comparable to the correlations between the full CPSS and these scales (*r*  =  0.47, 0.60, and 0.50, respectively).

## Discussion

Using data from two populations in distinct cultural contexts, we demonstrated that IRT analysis methods could identify specific items to retain from each measure and which items could be removed, allowing the creation of shortened measures that performed comparable to the standard longer measures on internal consistency reliability, construct validity, and criterion validity. In Thailand, use of these shortened measures in outcome analyses resulted in comparable effect sizes and yielded the same study conclusions as the longer standard scales, illustrating the ability of this method to capture change, but with the potential to significantly reduce respondent burden. With the reduction in the number of items on each measure and the ability to do this across outcomes, we created psychometrically valid instruments that could be pragmatically used across multiple research and practice-based settings; these scales are potentially short enough for both clinical and research purposes.

In the current study, we used criteria to select the items based on our goal to create both screening and monitoring tools. Our criteria for selecting items included: (1) high discrimination; (2) location parameters that represented a wide range of the latent trait; (3) reliability of item responses; (4) overlap with items on other scales; and (5) clinical relevance and utility. While this has worked well in this study, other criteria could be used for different purposes. For example, if screening was the only purpose of a scale, one might select items that cover a restricted range of the latent trait with more reliability. By illustrating our process, others will be able to understand how to use this method to create scales that will better serve their desired purpose.

Our research draws on trial and associated data related to task-shifted psychotherapy interventions (van Ginneken *et al*., 2013; Rathod *et al*., 2017; Seidman and Atun, 2017) to demonstrate that the short and long scales are comparable. However, the implications of the findings and the IRT process go beyond trials and task-shifted psychotherapy interventions. These methods can also inform surveys and program monitoring and evaluation. Normally in monitoring and evaluation of programs, monitoring consists of short repeated measures to monitor the process and longer measures to assess the impact of programs (Bolton *et al*., 2014; Kwan and Rickwood, 2015; Murray *et al*., 2015; Weiss *et al*., 2015). However, this approach is often not possible outside of a research context, as long assessment batteries cannot be feasibly implemented or sustained in routine practice, leading to little to no use of valid measures after the conclusion of a research study.

With these methods, we may be able to generate data that are both clinically useful and helpful in evaluating a program thereby negating the need for the longer measures of impact. In survey research, use of psychometrically valid short scales may give us a better understanding of how symptoms of psychopathology change in the absence of intervention – contributing to our understanding of the very nature of these disorders and how to better measure them going forward. Indeed, any study of multiple needs or multiple outcomes using standard length instruments carries the concerns of reduced cooperation and accuracy due to response fatigue (Diehr *et al*., 2005). Reducing instrument length while retaining accuracy is not only critical for repeated measures administration, but even for instruments that are used infrequently.

The development of valid, brief measures has additional utility beyond the typical uses as screening and outcome measures. The growth of transdiagnostic treatments worldwide provides an opportunity for brief measures to be integrated into the provision of care itself. Psychotherapy broadly, and the field of global mental health more specifically, is increasingly moving toward an intervention delivery system based on a common elements, or transdiagnostic, treatment approach (Farchione and Bullis, 2014; Murray *et al*., 2014; Newby *et al*., 2015; Gutner *et al*., 2016; Barlow and Farchione, 2017). A modular, multi-problem transdiagnostic approach that is designed to train providers in common elements that exist across a number of evidence-based mental health treatments (e.g. cognitive-behavioral therapy, cognitive processing therapy, interpersonal therapy) to provide them with the knowledge and skills to manage a range of common mental health and psychosocial problems, comorbidities and severities, thereby removing the ‘silos’ that exist for the treatment of individual disorders within mental health care (Chorpita *et al*., 2005; Murray *et al*., 2014; Murray and Jordans, 2016).

One of the key challenges with training non-specialist mental health providers in LMIC in transdiagnostic approaches is how to teach clinical decision making (i.e. what elements to give, in what order, and for how long). In LMIC, non-specialist providers may lack the training and depth of knowledge to make clinical judgments about the sequencing and dosage of the evidence-based therapeutic elements that are part of modular, multi-problem transdiagnostic approaches. Research on the CETA that has been developed and studied specifically for LMIC (Bolton *et al*., 2014; Weiss *et al*., 2015) has utilized a measurement-based care model (MBC), in which short, routine symptom measurement is used to inform how the treatment is provided. Short, frequently administered symptom assessments can inform the lay provider about the status of the client, which areas have improved, and which areas are in need of more attention (i.e. additional components or dosing). MBC across a number of psychotherapy approaches has been found to improve client clinical outcomes, increase engagement in care, and reduce the likelihood of treatment failure while also improving the provider's ability to track client progress (Eisen *et al*., 2000; Lambert *et al*., 2006; Morris and Trivedi, 2011; Scott and Lewis, 2015).

Measurement-based care based on brief psychometrically sound assessments also allows us to better understand how interventions are working and their efficacy in addressing a range of symptoms over time. For instance, measurement of symptoms at each session enables examination of longitudinal symptom trajectories and whether accelerated improvement in symptom severity is associated with the delivery of certain therapeutic elements (e.g. Sauer-Zavala *et al*., 2017). With this information, the mental health field can start to generate empirical evidence related to ‘critical ingredients’ or ‘mechanisms of action’ of interventions – what elements of interventions contribute to changes in outcomes – informing future dissemination, implementation, and scale-up of effective programs.

We are now using this approach in an RCT of a psychotherapy intervention in Ukraine (Murray *et al*., 2018*a*). Leveraging data collected as part of an instrument validation study (Doty *et al*., 2018), using IRT we were able to take a 123-item instrument that measured depression, PTS, anxiety, alcohol abuse, and impaired functioning and reduce it to 28-items (87% reduction in length). The resulting scales have comparable reliability and validity as the longer instruments (Doty *et al*., 2018). These 28 items guide delivery of treatment and have become our primary study outcome (Murray *et al*., 2018*a*). Using data from these 28 items across both intervention and control participants we will be able to see how people change in treatment and better understand the impact of individual structural elements of CETA.

### Limitations

These were secondary data analyses of existing data that were collected for treatment-based research purposes. Thus, our sample sizes were not specifically designed with IRT in mind. Our sample sizes are consistent with other IRT literature focused on scale refinement (i.e. 100–200) (Lincare, 1994; Marshall and Edelen, 2002). However, with our sample sizes, our item parameter estimates and scores might have large standard errors – an issue that is important for score calibration, but less of an issue for scale refinement (Edelen and Reeve, 2007). Another limitation is the potential that our analysis yielded results that fit the current data well but would not predict future observations reliably (i.e. over-fitting). We attempted to minimize this effect by splitting our data into development and testing samples – selecting items based on the development sample and then testing the shortened scale one time in the test data. Finally, the Zambia RCT cited in this paper was not yet completed upon publication; therefore, it was not possible to measure differences in effect sizes with the original and shortened versions of the CPSS.

## Conclusions

Our results illustrate the utility of IRT analytic methods for shortening mental health symptom measures across very different contexts and populations. Using these methods, we were able to create more concise measures for two mental health outcomes with results comparable to the standard, longer measures. In Zambia, we demonstrated how IRT can create reliable screening measures that accurately discriminate between psychosocial cases and non-cases. In Thailand, this same approach yielded similar evaluation results. This approach can be applied to the assessments for non-mental mental health as well. Using shortened measures has the potential to greatly reduce respondent burden providing more accurate information that can be used for both clinical and research purposes and provide the basis for a measurement-based care approach.

## Supplementary material

For supplementary material accompanying this paper visit http://dx.doi.org/10.1017/gmh.2019.30. click here to view supplementary material
