# Supplementary material for: When less is more: reducing redundancy in mental health and psychosocial instruments using Item Response Theory
Source: Glob Ment Health (Camb). 2020 Jan 9;7:e3. doi: 10.1017/gmh.2019.30 (PMC7003529; doi:10.1017/gmh.2019.30)
Supplement: Supplementary file 1 [file S205442511900030Xsup001.docx]

| Supplemental Table 1  Comparison of methods for each sample analyzed | | |
| --- | --- | --- |
|  | Thailand | Zambia |
| Sample origins | Screening, baseline, and follow-up data from an RCT | Instrument testing study |
| Sample Size | N = 653 | N = 210 |
| IRT Model | GRM | GRM |
| Reliability Metrics | Internal consistency reliability | Internal consistency reliability |
| Validity Metrics | Construct validity | Criterion Validity, Construct Validity |
| Sensitivity to Change | Intervention effect sizes | N/A |

| Supplemental Table 2  Thailand item parameter results (HSCL-25) | | | | |
| --- | --- | --- | --- | --- |
| **Item** | ***IRT Parameter*** | | | |
|  | ***a*** | ***b*_1_** | ***b*_2_** | ***b*_3_** |
| d01 | 1.58 | -0.55 | 0.48 | 1.95 |
| d02 | 1.56 | -0.14 | 0.88 | 2.66 |
| d03 | 2.9 | -0.82 | 0.15 | 1.68 |
| d04 | 1.16 | -0.35 | 1.14 | 2.72 |
| d05 | 1.03 | 0.91 | 1.96 | 3.97 |
| d06 | 1.8 | -0.01 | 0.75 | 2.47 |
| d07 | 1.51 | -0.28 | 0.97 | 3.09 |
| d08 | 1.2 | -0.03 | 1.54 | 3.81 |
| d09 | 1.41 | -1.04 | 0.13 | 2.26 |
| d10 | 1.47 | 1.64 | 3.12 | 4.57 |
| d11 | 2.28 | -1.23 | 0.07 | 1.62 |
| d12 | 2.41 | -0.62 | 0.31 | 1.89 |
| d13 | 2.02 | -0.28 | 0.57 | 2.06 |
| d14 | 1.24 | -0.21 | 0.98 | 3.51 |
| d15 | 1.54 | 0.55 | 1.45 | 2.81 |
| d16 | 2 | 0.03 | 1.08 | 2.62 |
| d17 | 2.85 | -1.19 | 0.17 | 1.61 |

| Supplemental Table 3  Thailand item parameter results (HTQ) | | | | |
| --- | --- | --- | --- | --- |
| **Item** | ***IRT Parameter*** | | | |
|  | ***a*** | ***b*_1_** | ***b*_2_** | ***b*_3_** |
| htq1 | 1.78 | -0.8 | 0.18 | 1.97 |
| htq2 | 2.13 | 0.43 | 1.18 | 2.62 |
| htq3 | 0.9 | 0.33 | 1.93 | 4.78 |
| htq4 | 1.87 | 0.1 | 1.04 | 2.52 |
| htq5 | 1.7 | -0.18 | 0.77 | 2.27 |
| htq6 | 1.36 | 0.65 | 1.66 | 4.78 |
| htq7 | 1.39 | -0.08 | 0.96 | 2.98 |
| htq8 | 1.53 | -0.14 | 0.91 | 2.97 |
| htq9 | 2.07 | -0.6 | 0.29 | 1.89 |
| htq10 | 1.45 | -0.07 | 1.33 | 3 |
| htq11 | 1.54 | -0.41 | 0.54 | 2.39 |
| htq12 | 0.96 | 0.44 | 1.83 | 4.74 |
| htq13 | 1.95 | 0.33 | 1.19 | 3.12 |
| htq14 | 1.28 | 0.45 | 0.57 | 3.78 |
| htq15 | 1.2 | -0.85 | 0.79 | 2.56 |
| htq16 | 1.24 | -0.99 | 0.53 | 2.96 |
| htq17 | 1.88 | -0.45 | 0.72 | 2.29 |
| htq18 | 1.39 | 0.1 | 1.58 | 3.41 |
| htq19 | 2.18 | 0.12 | 0.79 | 2.04 |
| htq20 | 2.16 | 1.08 | 1.8 | 2.96 |
| htq21 | 1.31 | -0.14 | 1.23 | 3.45 |
| htq22 | 1.68 | 0.72 | 1.47 | 3 |
| htq23 | 1.82 | 0.39 | 1.49 | 3.27 |
| htq24 | 1.83 | 0.86 | 1.71 | 3.1 |
| htq25 | 1.7 | 0.36 | 1.4 | 2.8 |
| htq26 | 1.37 | 0.47 | 1.82 | 3.75 |
| htq27 | 2 | 0.16 | 0.95 | 1.95 |
| htq28 | 1.45 | 1.03 | 2.53 |  |
| htq29 | 1.48 | 2.07 | 3.13 | 4.01 |
| htq30 | 0.97 | 0.38 | 1.51 | 3.56 |

| Supplemental Table 4  Zambia item parameter results (CPSS) | | | | |
| --- | --- | --- | --- | --- |
| **Item** | ***IRT Parameter*** | | | |
|  | ***a*** | ***b*_1_** | ***b*_2_** | ***b*_3_** |
| CPSS16 Bad dreams/nightmares | 1.78 | -0.79 | 0.79 | 1.24 |
| CPSS17 Felt like event happening again | 2.24 | 0.16 | 1.08 | 1.44 |
| CPSS18 Upset thinking or hearing about event | 2.42 | 0.20 | 0.89 | 1.22 |
| CPSS19 Feelings in your body when thinking of event | 3.15 | -0.04 | 0.76 | 1.06 |
| CPSS20 Avoid talking or thinking about event | 2.37 | 0.14 | 0.94 | 1.4 |
| CPSS21 Avoid activities, people, places that remind of event | 2.37 | -0.02 | 0.76 | 1.04 |
| CPSS22 Unable to remember parts of the event | 2.56 | -0.01 | 0.79 | 1.18 |
| CPSS23 Less interest in things | 2.71 | 0.07 | 0.76 | 1.16 |
| CPSS25 Unable to have strong feelings | 2.10 | -0.17 | 0.85 | 1.20 |
| CPSS26 Feel like future plans/hopes will not come true | 2.43 | -0.15 | 0.78 | 1.14 |
| CPSS27 Trouble falling or staying asleep | 1.97 | 0.37 | 1.34 | 1.79 |
| CPSS28 Irritable/fits of anger | 1.88 | -0.37 | 0.98 | 1.41 |
| CPSS29 Trouble concentrating | 2.44 | -0.12 | 0.86 | 1.31 |
| CPSS31 Jumpy easily startled | 2.21 | -0.14 | 1.09 | 1.66 |
